# A Re-Examination of “Bias” in Human Randomness Perception

**DOI:** 10.1037/xhp0000462

**Published:** 2017-10-23

**Authors:** Paul A. Warren, Umberto Gostoli, George D. Farmer, Wael El-Deredy, Ulrike Hahn

**Affiliations:** 1 Division of Neuroscience and Experimental Psychology, School of Biological Sciences, Faculty of Biology, Medicine, and Health, Manchester Academic Health Science Centre, University of Manchester; 2 Division of Neuroscience and Experimental Psychology, School of Biological Sciences, Faculty of Biology, Medicine, and Health, Manchester Academic Health Science Centre, University of Manchester, and School of Biomedical Engineering, University of Valparaiso, Chile; 3 Department of Psychological Sciences, Birkbeck University of London

**Keywords:** cognitive bias, perception of randomness, gambler’s fallacy

## Abstract

Human randomness perception is commonly described as biased. This is because when generating random sequences humans tend to systematically under- and overrepresent certain subsequences relative to the number expected from an unbiased random process. In a purely theoretical analysis we have previously suggested that common misperceptions of randomness may actually reflect genuine aspects of the statistical environment, once cognitive constraints are taken into account which impact on how that environment is actually experienced (Hahn & Warren, *Psychological Review*, 2009). In the present study we undertake an empirical test of this account, comparing human-generated against unbiased process-generated binary sequences in two experiments. We suggest that comparing human and theoretically unbiased sequences using metrics reflecting the constraints imposed on human experience provides a more meaningful picture of lay people’s ability to perceive randomness. Finally, we propose a simple generative model of human random sequence generation inspired by the Hahn and Warren account. Taken together our results question the notion of bias in human randomness perception.

## Public Significance Statement

The dominant perspective in experimental psychology is that human judgment and decision making are flawed. This is particularly evident in research on human perception of randomness. Here we explore this idea, presenting several analyses of data from an experiment in which participants are asked to generate a sequence of outcomes from a binary random process (like a coin toss). Although behavior does depart from the output of genuinely random source, the extent of this departure depends on how performance is characterized and whether constraints on human memory and attention span are taken into account. We find that when such constraints are considered, and appropriate performance measures are used, humans actually match the random source rather well. We argue more generally it may be problematic to emphasize errors in human judgment and decision-making without taking account of appropriate constraints.

Randomness is the flip side of statistical structure. Consequently, researchers interested in human beings as “intuitive statisticians” have long been interested in people’s ability to identify patterns of data as random. A long tradition of research has reached rather negative conclusions about people’s intuitive understanding of randomness. Whereas early studies focused primarily on people’s ability to generate random sequences (see, e.g., Wagenaar, 1972), later work has also examined people’s ability to judge sequences as random (see, e.g., Bar-Hillel & Wagenaar, 1991; Kahneman & Tversky, 1972; and see Oskarsson, Van Boven, McClelland, & Hastie, 2009 for an extensive review).

Both studies of sequence generation and production have found evidence of similar biases, in particular a bias toward overalternation between the different possible outcomes, such as “heads” (H) or “tails” (T), in binary sequences. This alternation bias has frequently been interpreted as evidence for a belief in the “gambler’s fallacy” (GF), that is, the erroneous belief that an increasing run of one outcome (e.g., HHHHHH . . .) makes the other outcome ever more likely (but see, e.g., Edwards, 1961).^1^ Such a belief, which can indeed be found among gamblers around the world (Clotfelter & Cook, 1993; Croson & Sundali, 2005; Terrell, 1998; Toneatto, Blitz-Mille, Calderwood, Dragonetti, & Tsanos, 1997), may reflect a mistaken conception of random processes as “self-correcting” in such a way as to maintain an equal balance between the possible outcomes (for other explanations see, e.g., the review of research on the GF by Hahn, 2011).

However, the concept of randomness is a difficult, and often counterintuitive, one not just for gamblers or experimental participants, but also for experimenters (on the concept of randomness see, e.g., Beltrami, 1999), and extensive critiques have shown much of the empirical research on lay understanding of randomness to be conceptually flawed (see in particular, Ayton, Hunt, & Wright, 1989; Nickerson, 2002; but also Lopes, 1982). Aforementioned evidence from real-world gamblers aside, it is less clear than might be expected how good or bad lay people’s ability to both discern and mimic the output of random sources actually is.

Research with novel tasks, that do not suffer from the conceptual flaws identified, have tended to confirm some element of bias in people’s performance (e.g., Olivola & Oppenheimer, 2008; Rapoport & Budescu, 1992) while finding also that participants’ performance is considerably better than deemed by past research (see, e.g., Lopes & Oden, 1987; Nickerson & Butler, 2009).

In particular, it has been argued that people’s performance may actually be quite good given their actual experience of random sequences, whether inside or outside the lab. Williams and Griffiths (2013) show how seemingly poor performance on randomness judgment tasks may stem from the genuine paucity of the available statistical evidence. Hahn and Warren (2009) similarly argue that common biases and misperceptions of randomness may actually reflect genuine aspects of the statistical environment, once it is taken into account how that environment is actually experienced. Specifically, Hahn and Warren demonstrate that if human experience of a stream of binary random events is assumed to be (a) finite and (b) constrained by the limitations of short-term memory (STM) and/or attention, then based upon highly counterintuitive mathematical results, not all binary substrings are equally likely to occur.

We next describe this theoretical account in more detail, before going on to present the results of two behavioral experiments that provide evidence that human perception of randomness conforms to the theoretical treatment outlined. Finally, we present a simple generative model of human random sequence generation that reflects key features of the Hahn and Warren account.

## Hahn and Warren (2009) Account of Randomness Perception

The theoretical account of randomness perception in Hahn and Warren (2009, 2010) relies upon a simple model of how a human might experience an unfolding sequence of random events. It is proposed that humans have a limited capacity *window of experience* of length *k* that has access to the present event and preceding *k*-1 events. This window slides one event at a time through an unfolding finite sequence of length *n* > *k*. That humans could only ever experience a finite stream of events is incontrovertible. Further, given the well-characterized bounds on human STM capacity and/or attention span, this limited capacity, sliding window of experience account seems plausible.

**Figure 1 fig1:**
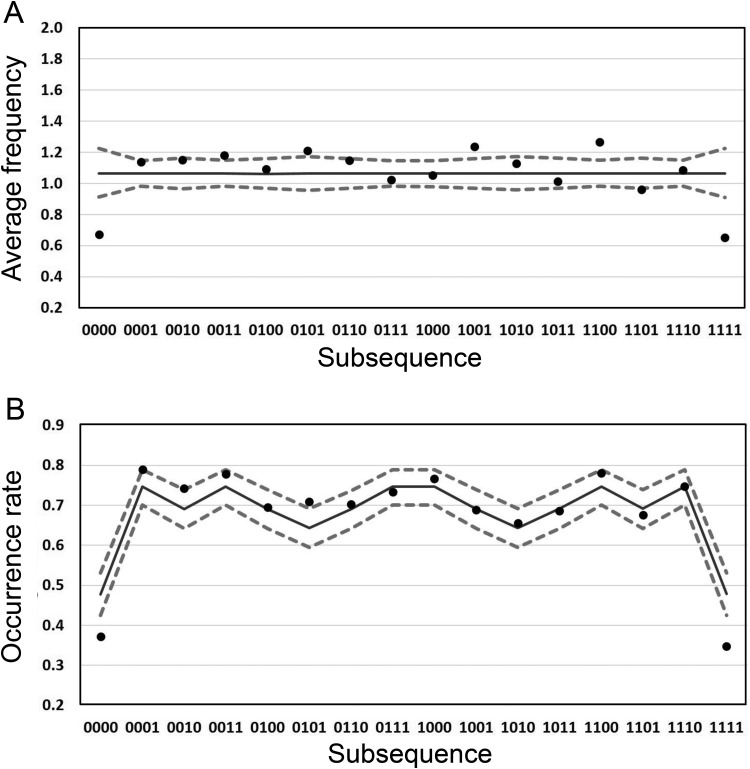
(A) Results of Analysis 1 for sliding window length 4. Average subsequence frequencies per 20-bit block are presented for both human-generated (dots) and the theoretically unbiased (TU) data (solid line, 95% confidence interval [CI] dashed lines). (B) The results of Analysis 2 for sliding window length 4. Proportions of 20-bit blocks containing at least one occurrence of the subsequence are presented for both human-generated (dots) and TU data (solid line, 95% CI dashed lines).

Crucially, when subsequences of length *k* are counted among a longer finite sequence of length *n* using the sliding window analysis suggested above, certain subsequences are more likely to not occur, *even when the generation process is unbiased*. In particular perfect runs of one outcome have highest *nonoccurrence probability* (or conversely lowest occurrence rate), followed by perfect alternations of the two outcomes. This highly counterintuitive mathematical result is illustrated in Figure 1B; the unbroken line represents the occurrence rates for the 16 possible subsequences of length 4. For example, the occurrence rate for the perfect run subsequence 0000 is around 0.47 meaning that this subsequence does not appear at all on around 53% of all sequences of length 20 generated by an unbiased random process. In contrast the occurrence rate for subsequence 0001 is around 0.75 meaning that this subsequence does not appear on only around 25% of unbiased sequences of length 20. Hahn and Warren (2009) argue that if human experience of unfolding random events mimics the sliding window, then this could explain three key tendencies of human randomness perception that are taken as evidence of bias:[Fig-anchor fig1]

1 A tendency to think that sequences with some irregularity are more likely given an unbiased coin.
2 An expectation of equal numbers of heads and tails within a sequence.
3 A tendency to overalternate between outcomes when generating random sequences.

Based on theoretical data of the kind presented here (Figure 1B unbroken line), Hahn and Warren argue that (a) is reasonable, that is, the figure demonstrates that there is statistical support for the intuition that regular subsequences (e.g., 1111, 0101) occur less often than irregular subsequences (e.g., 0100, 1101). Hahn and Warren also argue that (b) is consistent with the sliding window account because it is difficult to distinguish between the vast majority of sequences using occurrence rate (Figure 1B, unbroken line) suggesting judgments should be based not on an explicit coding of each subsequence but something simpler such as the proportion of heads. Finally, Hahn and Warren argue (c) follows directly from the sliding window account because short sequences tend to have more alternations between outcomes than expected in an infinite series (Kareev, 1992).

## Overview

In the present study we examine the characteristics of human randomness perception in light of the theoretical account of Hahn and Warren (2009) across two experiments. Although a range of tasks have been used previously to investigate randomness perception, sequence generation has been by far the most dominant and, accordingly, we use this task in both our experiments. In Experiment 1 we asked participants to first observe the output of a random source before generating a random binary sequence. In Experiment 2 we replicated Experiment 1 but also examined the effect of recent experience by comparing sequences generated both before and after exposure to the random source. To preempt our results, in both experiments we find that when compared on *expected frequency of occurrence* of binary subsequences, behavior departs markedly from that of an unbiased random generating process. This is a common finding in the literature and such results give rise to the notion of bias in randomness perception, since for an unbiased random process the expected frequencies should all be equal for any specified subsequence length. However, we also show that human sequences are remarkably similar to those of an unbiased random generation process when other methods of comparison are used that are relevant to the sliding window account (e.g., subsequence occurrence rate or direct comparison of subsequence frequency distributions for a given window length), and that this is particularly evident at subsequence lengths around 4. This is a plausible length of the typical human window of experience as defined above and in line with research suggesting that the effective span of STM is 4 when strategies such as rehearsal are ruled out (Cowan, 2001, 2010). Finally, we present a simple model of binary sequence generation in humans that incorporates the key features of the Hahn and Warren (2009) account. This model generates binary outcomes with one free parameter, reflecting the extent to which the probability of runs of the same outcome (e.g., 111...1) is down-weighted to favor sequences in which the run is ended (e.g., 111...0).

## Experiment 1

Participants first observed blocks of binary outcome random sequences following an unbiased Bernoulli process (*p* = .5) and were then instructed to generate random outputs to match the properties of the observed process.

### Method

#### Participants

Twelve undergraduate students from the University of Manchester participated on a voluntary basis and gave informed consent. Participants received course credit as payment. There were no exclusion criteria.

#### Materials

Participants were seated in front of a 19-inch LCD display. The experimental stimuli were presented using the Python programming language on a PC running Windows 7. Participants responded using a standard Windows keyboard.

#### Procedure

Participants were told they would first observe the output of a machine generating a random sequence of 1’s and 0’s, and that they should attend to it (Presentation Phase) before going on to generate a sequence (Generation Phase).

Presentation Phase: Each digit (a 1 or 0) appeared on the screen for 250 ms before being replaced by the next digit in the sequence. The display of each digit was accompanied by a corresponding tone. The display was full screen with a black background. The digits were displayed in white in 80 point Arial font in the center of the screen. To reinforce the signal provided by the random source 1’s were accompanied by a high (1200 Hz) tone, and 0’s by a low (800 Hz) tone.^2^ After every 20 digits the sequence paused and participants were required to complete a distractor task. The distractor task consisted of counting the number of vowels in a list of 10 words. In total participants observed 600 digits over 30 blocks of length 20.

Generation Phase: Participants were asked to generate a new sequence representative of the one they had just observed in the Presentation Phase. They used the keyboard to press either 1 with their left hand, or 0 with their right hand. For each key press participants saw the appropriate digit on screen and heard the corresponding tone, exactly as in the presentation phase. As in the Presentation Phase, participants generated 600 digits in 30 blocks of 20 and the same distractor task was used in between each block.

#### Data analysis

We compared the statistical properties of sequences generated by a truly random Bernoulli process (*p* = .5) and those generated by our participants (*N* = 12). Based on evidence that the effective span of short term memory is 4 items, when strategies such as rehearsal are ruled out (Cowan, 2001, 2010), we describe our analysis, and present results for *k* = 4 only. However, we have repeated our preliminary analyses for other values *k* = 3 to *k* = 6 (see supplemental materials). For each participant, and each of the 30 blocks of data collected, we slid a window of length *k* = 4 through the 20-bit sequence of generated outcomes. We then undertook four analyses of these sequences by aggregating data across observers. From 12 participants generating 30 × 20-bit sequences we had 360 sequences over which to assess performance. We undertook the following four analyses to characterize performance in different ways.

**Analysis 1:** We calculated the average observed frequency for each of the 16 possible subsequences per 20-bit sequence. Note that for an unbiased random process the expected frequency of each subsequence should be 1.0625 per 20-bit sequence. When randomness perception is referred to as biased, it is typically based on the observation that participant generated subsequences do not occur with equal frequency (e.g., alternating sequences are overrepresented and runs are underrepresented).

**Analysis 2:** We calculated the *occurrence rate*— that is, the proportion of 20-bit sequences that contained ***at least one*** occurrence of each of the 16 possible subsequences. Note this metric is the complement of the *nonoccurrence probability* described by Hahn and Warren (2009). Even for an unbiased random process this metric will not be the same for all subsequences (see Hahn & Warren, 2009 and Figure 1B).

**Analysis 3:** We generated histograms illustrating the proportion of 20-bit sequences containing 0, 1, 2, and so forth . . . occurrences of three subsequences AAAA, ABAB, AAAB (averaged over A = 1, B = 0 cases and vice versa) that are particularly interesting under the Hahn and Warren (2009) account. Subsequence 0000 (and its complement 1111) has special status since its nonoccurrence rate for plausible values of *n* and *k* is markedly different from the other sequences. Similarly, subsequence 0101 (and its complement 1010) is interesting because its nonoccurrence rate is lower than the other sequences. Subsequence 0001 (and its complement 1110) is interesting when compared to a perfect run of the same length. This comparison is relevant to the gambler’s fallacy phenomenon. Note that Analysis 1 is equivalent to calculating the expected value of such distributions for each of the 16 subsequences.

**Analysis 4:** The histograms generated in Analysis 3 contain significant positive skew. Consequently we generated boxplots illustrating the median, Inter-Quartile Range (IQR) and extreme data for the distributions obtained in Analysis 3.

We also generated the same amount of data (360 × 20-bit sequences) as that obtained from human participants from a genuinely unbiased Bernoulli process (*p* = .5). We refer to these simulated sequences as the theoretically unbiased (TU) data-set and their properties are analyzed in an identical manner to the human data. By repeatedly generating (*N* = 1,000) TU data-sets we were able to place confidence bounds on the metrics described in Analysis 1 and 2 for a TU participant.

### Results

In Figure 1A the dots represent the observed expected values of human-generated subsequence frequencies (Analysis 1) at window length 4. The unbroken black lines represent the equivalent metric for the TU participant. The dashed lines represent the 95% confidence interval (CI) on the TU data. Note that the TU expected frequencies are the same across subsequences since in an unbiased random process all subsequences at all window lengths should be equally represented (e.g., see Beltrami, 1999). Although the majority of the human data lies within the CI for the TU data, there are some clear departures and there appears to be systematic over- and underrepresentation of certain subsequences relative to the TU data. This analysis illustrates the standard description of human random sequence generation as biased. Relative to the TU data, the perfect runs are clearly underrepresented and 10 of the other 14 subsequences are overrepresented.

Figure 1B shows the outcome of Analysis 2 for window length 4. The dots represent the occurrence rate—that is, the proportion of the 360 blocks on which a subsequence occurred at least once—for human participants. Respectively, the solid black and dashed lines illustrate the equivalent occurrence rate and 95% CI for the TU data. Using this analysis the human and TU data share several common features, including a marked decrease in occurrence rate for perfect runs. In addition the human data appear to follow the fluctuations in the TU data with high correlation between the sequence occurrence rates (*r* = .971).

**Figure 2 fig2:**
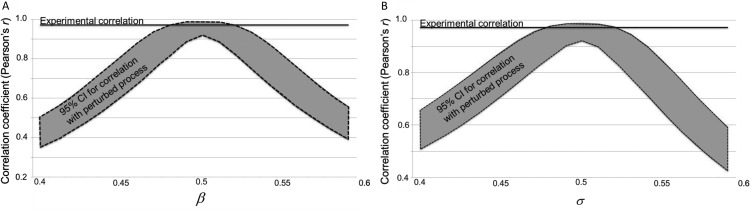
The results of the follow up analysis to examine degrees of correlation between occurrence rates of the observed human generated and theoretically unbiased (TU) subsequences (*k* = 4). (A) Variation in correlation between occurrence rates of an unbiased process and those that are biased in base rate (β). (B) Variation in correlation between occurrence rates of an unbiased process and those that are biased in Markov switching rate (σ).

We also undertook a follow-up analysis to further investigate the high correlation observed in Figure 2B. In particular, one might want to ask how remarkable it is to find such a high correlation and what degree of correlation might arise by mathematical necessity for any process that even crudely matches the properties of a genuinely random source. In other words, how closely does a generating source need to match a random process to give rise to the degree of distributional match observed in our data.[Fig-anchor fig2]

A simple thought experiment illustrates the issue. A truly random source has an expected long-term alternation rate of .5. This alternation rate could be matched perfectly by generating a sequence of perfectly alternating 0s and 1s (i.e., 0101010101 . . .). Though this sequence would match several of the statistical properties of sequences produced by random generating sources, it would fail to match the subsequence distribution statistics shown in Figure 1A and 1B.

In further analysis we examined the extent to which a random generating source would need to be perturbed away from unbiased to observe a marked drop in correlation in occurrence rates with those of a truly random process. We reasoned that if that correlation remains high over a large range of perturbations then the high correlation observed in our observers seems unremarkable. However, if the correlation is sensitive to small perturbations then it seems reasonable to suggest that the high correlation is because of genuine similarity between human observers and a random process and worthy of note. We perturbed the unbiased process in two ways:

1 By manipulating the base rate β, that is, the propensity of the source to generate 0’s and 1’s. Specifically, we changed the probability P(0) = β of generating a 0 on each step, and accordingly the probability P(1) = 1-β of generating a 1 on each step. Clearly for an unbiased random process β = 0.5. Increasing β above 0.5 leads to a bias toward producing 0’s whereas decreasing the parameter leads to a bias for 1’s.
2 By manipulating the switching rate σ of a Markov process, that is, the propensity of the source to transition from one possible state (0 or 1) at step *i* to the other state at step *i* + 1. Specifically, we defined a 2 × 2 Markov transition matrix M with diagonal entries reflecting the probability of sticking in the same state (0 or 1) set to 1-σ and off diagonal entries, reflecting the probability of switching (from 0 to 1 or vice versa) set to σ. For an unbiased random process σ = 0.5. Increasing σ above 0.5 leads to a bias toward switching whereas decreasing the parameter leads to a tendency to generate runs of the same outcome.

The 95% CIs for the correlation between the biased and unbiased generators as a function of the β and σ parameters are shown in Figure 2. Clearly the correlation coefficient obtained between the occurrence rates at window length four is rather sensitive to small perturbations away from a truly random process for both perturbation types. Therefore, we conclude that the degree of subsequence match observed in our data genuinely speaks to the degree of appreciation participants show for the characteristic outputs of random generating sources.

**Figure 3 fig3:**
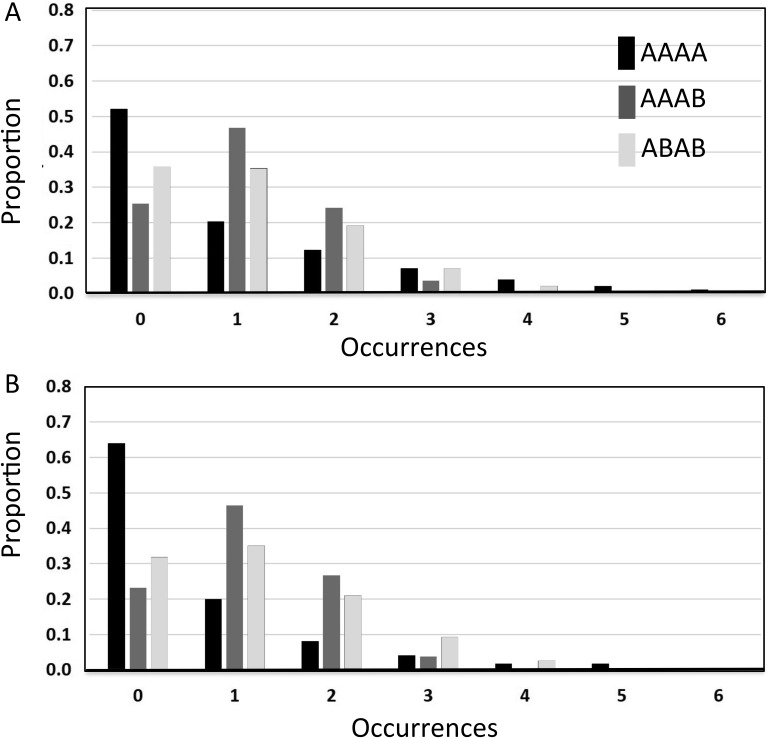
The results of Analysis 3 for sliding window length 4. Histograms describe the proportion of blocks containing each occurrence frequency for three selected subsequences. (A) theoretically unbiased (TU) data truncated at occurrence frequency = 6. Note the expected values of these three distributions are identical at 1.0625 (consistent with Analysis 1). (B) Data for human observers. Note that the expected values of these three distributions are different from 1.0625 and equal to the appropriate average frequency data points in Figure 1A.

As noted in Hahn and Warren (2010), although the nonoccurrence probability, or its complement the occurrence rate, is a convenient statistic with which to illustrate differences between subsequences it is not the only statistic for which differences emerge for an unbiased random process. In Analyses 3 and 4 we illustrate significant differences between the distributions, medians and modes of three key subsequence types: AAAA (i.e., 1111 and 0000), AAAB (i.e., 1110 and 0001), and ABAB (i.e., 1010 and 0101) and show that based on these analyses human and TU data are in close agreement. In Figure 3 we present the outcome of Analysis 3 for the TU (Figure 3A) and human (Figure 3B) data. Note, that occurrence rates obtained in Analysis 2 for the three subsequences considered can also be seen in Figure 3 as the sum of all columns except that for frequency 0. Although there are some differences in the human versus TU distributions they are primarily both qualitatively and quantitatively similar. Furthermore, the clear skew in the distributions of these data suggests that it might be problematic to use the expected value (i.e., the average number of occurrences calculated in Analysis 1) as a summary statistic. To reinforce this point note that the observed expected values of the three distributions in Figure 3B are given by the corresponding data points in Figure 1A. As noted in Analysis 1, for the human data these expected values are different. On the other hand for the TU data the expected values of the three distributions in Figure 3A are identical at 1.0625. However, considering the distributions, we see that the differences between human and TU data are actually rather subtle. For example, for the AAAA sequences, even though the expected value is considerably lower for human participants (around 0.7) than for the TU data distribution (1.0625) this discrepancy appears to be largely driven by the fact that high frequency sequences (e.g., beyond frequency 5) are underrepresented in the human data. These extreme values would contribute significantly to increasing the expected value even though they are highly unlikely to be experienced. As a consequence, we suggest that placing emphasis on the difference in expected values between human data and that generated by a TU process is problematic when there are similarities in the data generated on other (potentially more appropriate) statistics.[Fig-anchor fig3]

**Figure 4 fig4:**
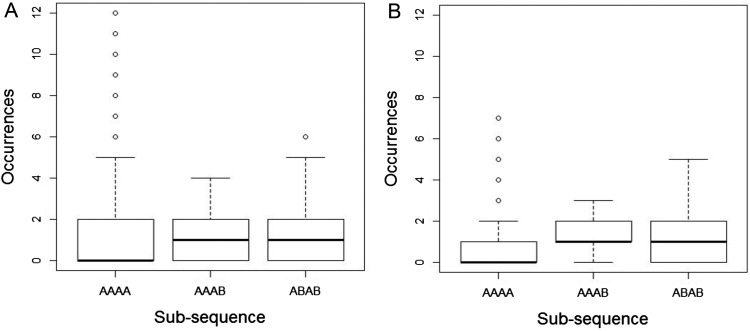
The results of Analysis 4 for sliding window length 4. Boxplots illustrating medians Inter-Quartile Range (IQRs) and extreme values of the data illustrated in Figure 2 for three selected sequences. (A) theoretically unbiased (TU) data (truncated at frequency = 12). (B) Data for human observers.

In Figure 4 we present another illustration of the data in Figure 3. These boxplots emphasize the similarity in the median frequency for the humans and TU data. In addition, box plots for the AAAB and ABAB subsequences are very similar between human and TU participants. Similar to Figure 3, for subsequence AAAA the increased tendency for the TU participant to generate high frequency sequences is also evident. As noted above, this tendency is responsible for the higher expected value for TU relative to human data. In addition we see that for an agent paying attention to the median statistic it would be true to say that subsequence AAAB is less likely to occur that AAAA. It is possible that this plays a role in the gambler’s fallacy.[Fig-anchor fig4]

Note that although we have focused exclusively on the analyses at window length *k* = 4 we have data for lengths from *k* = 3–6. We find that up to length 5 there is good correspondence between human and simulated data on Analyses 2, 3, and 4 but beyond this value the discrepancies are greatly increased^3^ (see supplemental materials for these analyses).

### Discussion

In this experiment we have provided preliminary evidence in line with the Hahn and Warren (2009) account of randomness perception. We showed that sequences generated by human participants were remarkably similar to those from a truly random process when compared on a set of metrics that are more appropriate given the constraints on how humans might actually experience random events.

One potential issue with this study is that we used a relatively small sample of participants. Arguably this makes our result even more surprising—we did not need large amounts of data to find similarities between our account and human data. However, it would be useful to replicate our results in a larger sample.

Furthermore, it is possible that the data generated by our participants after seeing a random source says more about ability of participants to mimic rather than their concept of randomness. To a certain extent this contention can be ruled out by showing that participant generated sequences are not well matched to the actual sequence observed. However, it would of course be more compelling to measure participants’ sequence generation behavior both before and after the random source experience. We will then be able to assess the extent to which participants’ perception of randomness was altered by that experience. If participants’ performance is altered by passively viewing a “machine generating a random sequence,” without any need to engage with the sequence (e.g., through outcome prediction as in Edwards, 1961), it would suggest both that experience of randomness is key, and that, consequently, the much bemoaned “biases” in randomness perception and generation are ultimately transient phenomena. This will be particularly compelling if the specific experience observed is not as well matched to human performance since this would suggest that participants have learned something general about random sequences rather than how to mimic a specific sequence. To investigate these issues we conducted a second experiment.

## Experiment 2

Experiment 2 was very similar to Experiment 1 with the following changes. We used ‘H’ and ‘T’ with the cover story of a fair coin, rather than ‘1’ and ‘0’, and whether or not participants heard a sound accompanying the visual stimuli was manipulated as a between subjects condition. The second difference was that participants were asked to generate a random sequence before being exposed to one. In the first experiment participants observed and then generated, in the second experiment participants generated, then observed, and then generated again. Experiment 2, therefore, allowed us to test for any learning that might occur from being exposed to a genuine random sequence. In all other respects Experiment 2 was identical to Experiment 1

### Method

#### Participants

Seventy-two participants from Birkbeck College, University of London were recruited and gave informed consent. Participants received £7.20 per hour as payment for their time. Participants had a mean age of 29 (*SD* = 11). There were 47 female participants and 25 male. There were no exclusion criteria.

#### Procedure

Participants first completed a generation task in which they were asked to produce a sequence representative of flipping a fair coin. Following the initial generation phase the experiment proceeded as in Experiment 1 with an observation and then generation phase. To investigate the possible moderating effects of the sounds used in Experiment 1, half of the participants in Experiment 2 did not hear an accompanying sound.

#### Analyses

From 72 participants generating 30 × 20-bit sequences we had 2,160 sequences per condition over which to assess performance. We conducted the same analyses as in Experiment 1 with the addition of a mixed 2 × 2 analysis of variance (ANOVA) to investigate the within subjects effects of generation period (pre, post), and the between subjects effect of an accompanying sound (silent, tones). The dependent variable was the Root Mean Square Error (RMSE) for the occurrence rate of each of the possible length-four subsequences and the expected occurrence rate under the Hahn and Warren account (analysis 2 in Experiment 1).

### Results

#### Replication of analyses from Experiment 1

**Figure 5 fig5:**
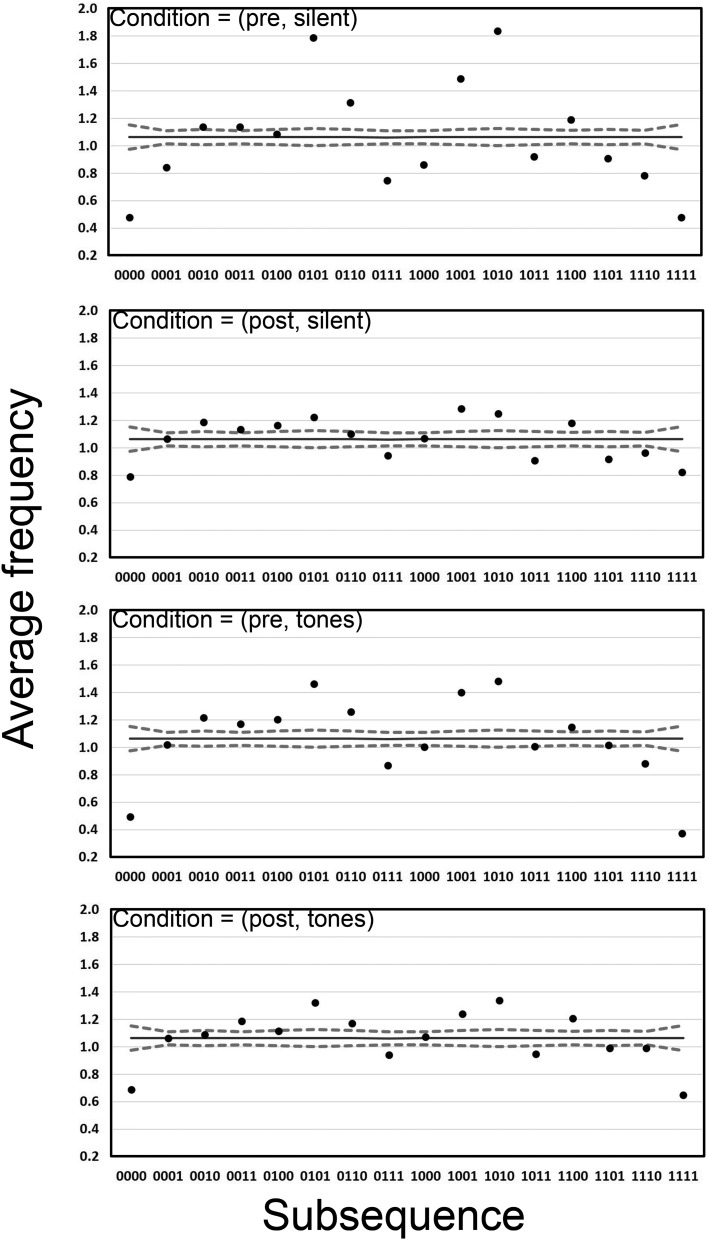
Analysis 1 for *k* = 4 in the four conditions of Experiment 2. Average subsequence frequencies per 20-bit block are presented for both human-generated (dots) and the theoretically unbiased (TU) data (solid line, 95% confidence interval [CI] dashed lines).

Average frequencies of each subsequence per 20-bit long generated sequence are shown in Figure 5.[Fig-anchor fig5]

Broadly speaking the data in Figure 5 are consistent with those presented in Figure 1A in that there are clear departures in average frequency from those expected from the TU data. Note that the data are similar irrespective of the tones condition but average frequencies appear closer to those of the TU data in the post condition.

**Figure 6 fig6:**
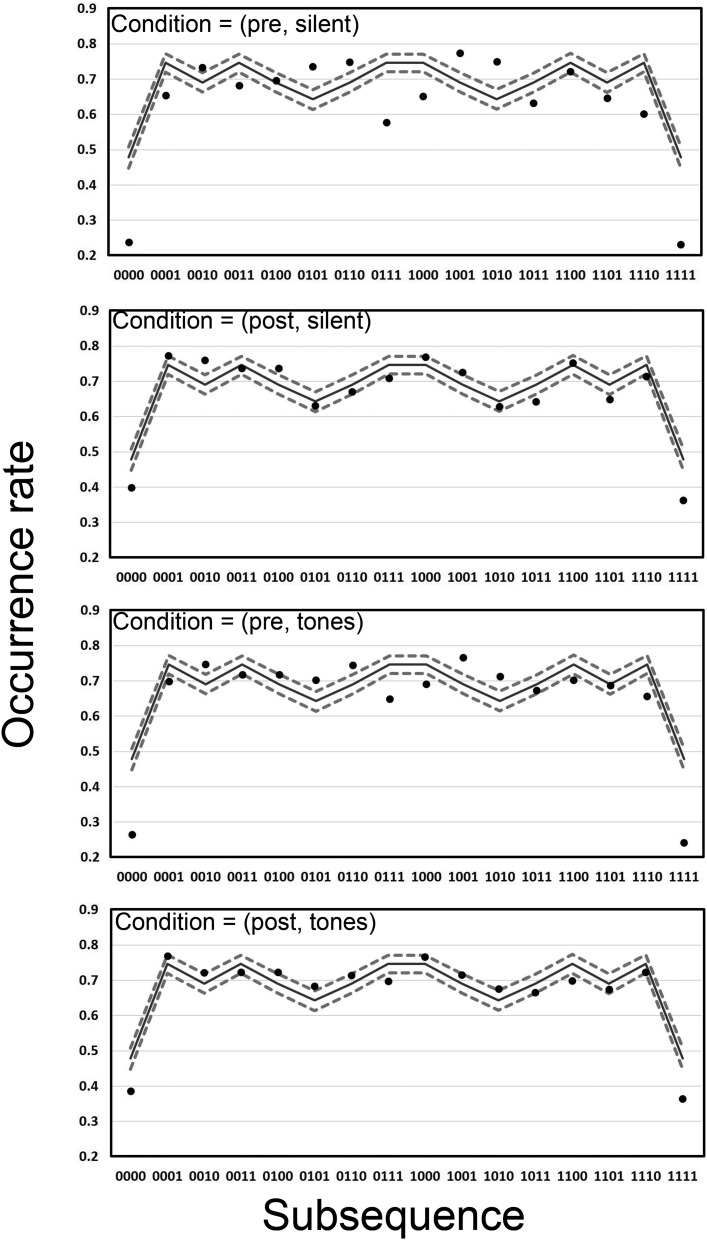
Analysis 2 for *k* = 4 in the four conditions of Experiment 2. Occurrence rates are presented for both human-generated (dots) and theoretically unbiased (TU) data for length 20 sequences (solid lines, with 95% confidence interval [CI] as dashed lines).

Figure 6 shows the occurrence rate for each subsequence per 20-bit long generated sequence in the four conditions of Experiment 2. Similar to the data in Figure 5, there is limited evidence of an effect of the tones factor on performance. Once again the data are in line with the results of Experiment 1. Consistent with the data from Experiment 1 in Figure 1B, when analyzed based on the occurrence rate metric the human and TU data are remarkably similar. This is particularly the case in the post conditions, suggesting that experience of a random source has lead to human sequence generation that is closer to the TU data.[Fig-anchor fig6]

**Figure 7 fig7:**
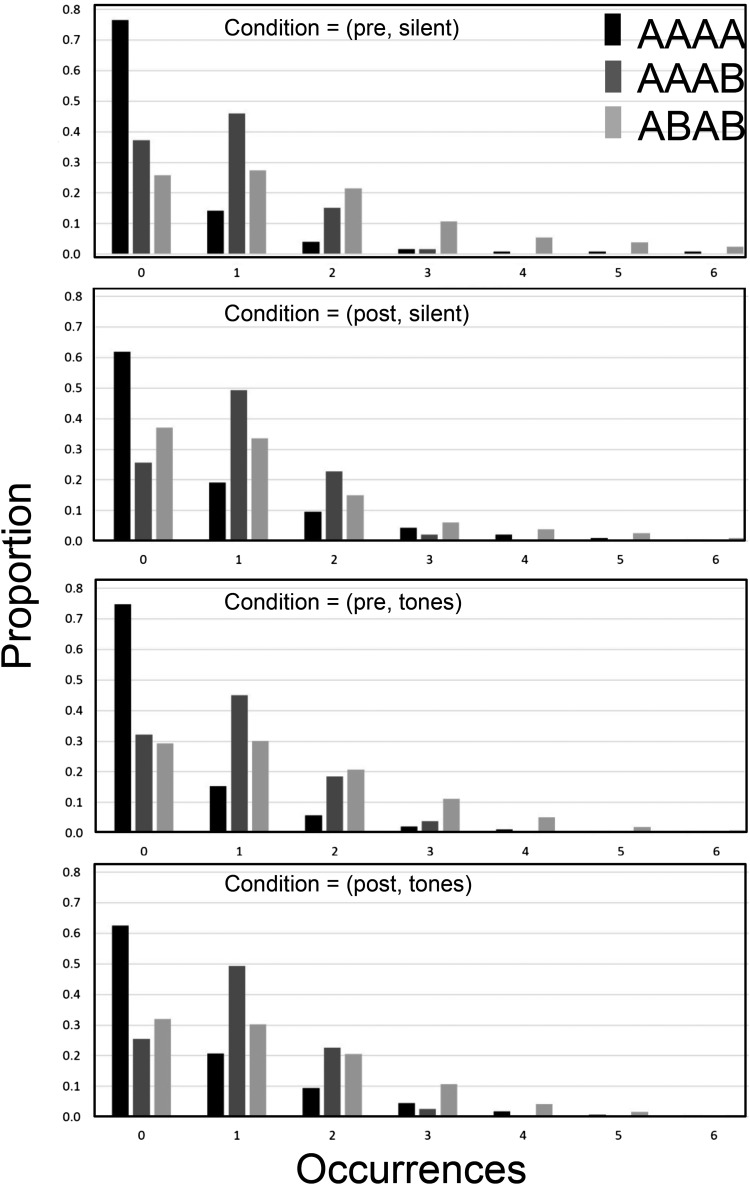
The results of Analysis 3 for sliding window length 4 for the four conditions in experiment 2. Histograms describe the proportion of blocks containing each occurrence frequency for three selected subsequences.

Figure 7 shows histograms of the proportion of times a 20-bit long sequence contained 0, 1, 2,.. occurrences for the three subsequences AAAA (i.e., 1111 and 0000), AAAB (i.e., 1110 and 0001), and ABAB (i.e., 1010 and 0101). The data are again similar to those obtained in analysis 3 of the data from Experiment 1 (see Figure 3). Note that as with Figures 5 and 6 there is evidence that exposure to the random source has affected performance and that the human-generated data are closer to the TU data (Figure 3B) in the post conditions (in particular note that the AAAA and AAAB bars for 0 occurrences are nearer to the values from the TU data in Figure 3B in the post conditions).[Fig-anchor fig7]

**Figure 8 fig8:**
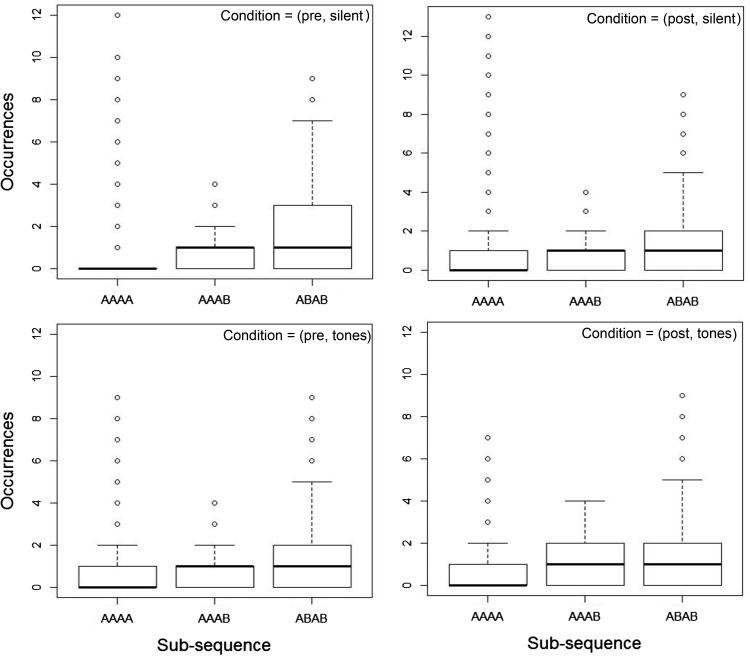
Analysis 4 for *k* = 4 in the four conditions of Experiment 2. Boxplots illustrate medians, Inter-Quartile Range (IQRs), and extreme values of the data for the three sequences types AAAA, AAAB, and ABAB.

Figure 8 shows the outcome of analysis 4 for the conditions in Experiment 2. Similar to the data obtained in Experiment 1 these boxplots emphasize the similarity in the median frequency for the humans and TU data (Figure 4A). Based on Figure 4A, for an agent paying attention to the median statistic it would be true to say that subsequence AAAB is less likely to occur that AAAA and this pattern of data emerges in the human generated sequences also.[Fig-anchor fig8]

#### Tests for differences between conditions

**Figure 9 fig9:**
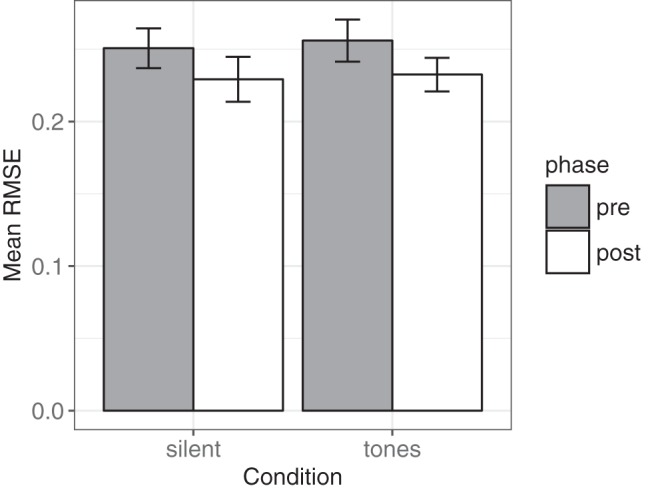
Mean root mean square error of approximation (RMSE) between Hahn and Warren account and participants’ generated sequences in each of the conditions. There was no significant effect of sound, but there was a significant reduction in RMSE after participants had observed a genuine random sequence. Error bars represent ±1 *SEM*.

A 2 × 2 mixed ANOVA tested the RMSE correspondence between the generated sequences and those expected under the Hahn and Warren (2009) account. Between subjects we manipulated sound (silent, tones) and within subjects we manipulated experience (pre, post). There was a significant main effect of experience *F*(1, 70) = 4.25, *p* = .043, η^2^ = 0.06, but not of sound *F*(1, 70) = 0.07, (*p* = .796). These results indicate that the participants’ generated sequences were better described by the Hahn and Warren account after observing a genuine random sequence (Mean RMSE = 0.23, *SD* = 0.08) than before (Mean RMSE = 0.25, *SD* = 0.08; Figure 9).[Fig-anchor fig9]

### Discussion

The results of Experiment 2 are broadly in line with those of Experiment 1 across Analyses 1–4. Replicating these findings with a much bigger data set (Exp. 1: *N* = 12 vs. Exp. 2: *N* = 72) rules out the possibility that the close correspondences observed in Experiment 1 between human and TU data on the metrics considered were because of having used a small sample size. In addition we have ruled out the possibility that our data were affected by the way in which the exposure to a genuinely random source was presented (i.e., purely based on visual vs. visual and auditory information).

**Figure 10 fig10:**
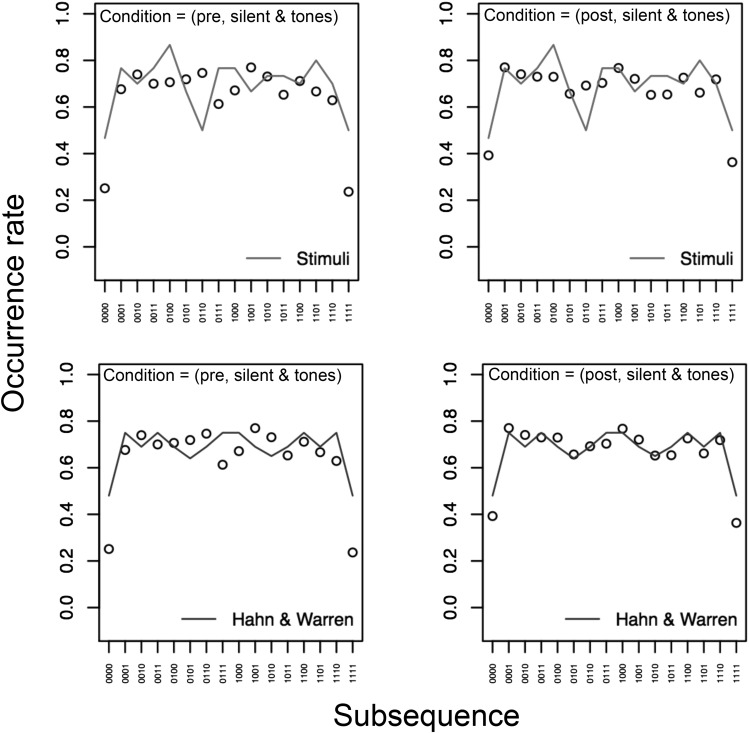
Human occurrence rate data (circles) in the pre- and postexperience conditions (averaged over the silent and tones conditions) observed in the four conditions of Experiment 2. In the top row we also show the occurrence rates that might be expected if the observer were trying to mimic the actual sequence observed (solid line). In the bottom row we also show the occurrence rates based on the Hahn and Warren (2009) account (solid line). Clearly the participant data is closest to the Hahn and Warren account occurrence rates and this is particularly true in the postexperience data. Postexperience and HW09 RMSE = 0.05. Postexperience and stimuli RMSE = 0.09 (RMSE = root mean square error).

With respect to the issue of whether our participants were simply mimicking sequences observed, we feel we can now argue strongly against this point. By comparing the pre- and postexposure conditions we see that our participants produced behavior that was indeed closer to that of a genuinely unbiased process after having experience of outputs from such a source. However, given that the properties of the specific experience observed are not well matched to human performance (see Figure 10) we conclude that participants have learned something general about random sequences rather than how to mimic a specific sequence.[Fig-anchor fig10]

## A Simple Generative Model of Binary Sequence Generation

What is it exactly that participants have learned? In this section we outline a simple generative model with one free parameter that closely approximates participant generated sequences. Inspired by the Hahn and Warren (2009) account, this model is generative in the sense that on each step a new binary digit is produced. The key characteristics of the Hahn and Warren account relevant for this model are: (a) that humans experience random events through a sliding window of experience of length *k* and (b) that behavior is largely driven by sensitivity to the difference between long runs and the other sequences, that is, the majority of subsequences are not distinguished by observers but perfect runs have a special status, because of the large difference in occurrence rate observed (see Figures 1B and 6) for TU sequences when *n* and *k* have plausible values.

The model starts by randomly generating *k*-1 binary digits to produce substring *s*_*i*_^-^ = [*d*_1_, *d*_2_, . . ., *d*_*k*-1_ ] where the *d*_*i*_ correspond to binary digits. To generate the next digit, *d*_*k*_, the model considers the possible length *k* subsequences that would result from the possible digit selections. Of course, given a binary alphabet there are only two such options, namely [*s*_*i*_^-^, 0] or [*s*_*i*_^-^, 1]. The model then selects one of these options, either *d*_*k*_ = 0 or *d*_*k*_ = 1 with probabilities *p*_*0*_ *or p*_1_ (= 1-*p*_*0*_) respectively, which results in the first length *k* substring *s*_1_. To implement the sliding window, that is, characteristic (a) above, this process then repeats so that on step *i*, *s*_*i*_^-^ = [*d*_*i*_, *d*_*i*+1_, . . ., *d*_*i+k*-1_] and *s*_*i*_ is either [*s*_*i*_^-^, 0] or [*s*_*i*_^-^, 1]. We propose a free parameter β that acts to “boost” or “de-boost” the relative probability of one outcome, *d*_*k*_ = 0 or *d*_*k*_ = 1, over the other on each step. For a genuinely random generation process β = 0.5. However, to implement characteristic (b) we suggest that the probability of an alternation after a run of the same outcome will be boosted. Specifically, *p*_1_ = β > 0.5 for *s*_*i*_^-^ = [0, 0 . . ., 0] and *p*_*0*_ = β > 0.5 for *s*_*i*_^-^ = [1, 1 . . ., 1].

We used the model to generate 100,000 20-bit sequences with a plausible window of experience length of 4 (Cowan, 2001, 2010; i.e., *k* = 4), for values of the boost parameter β varying from 0.0 to 1.0 in steps of 0.05. Based on these model-generated sequences we estimated the occurrence rate across repetitions for each length 4 subsequence and for each value of β. We could then interpolate the resultant look-up table to estimate the occurrence rate for each subsequence as a function of β. Using this interpolation scheme we then fitted (using the MatLab fminsearch algorithm) human data by adjusting the boost parameter for the complementary pair of subsequences associated with stopping long runs (i.e., boosting *p*_1_ after 000 and *p*_0_ after 111).

**Figure 11 fig11:**
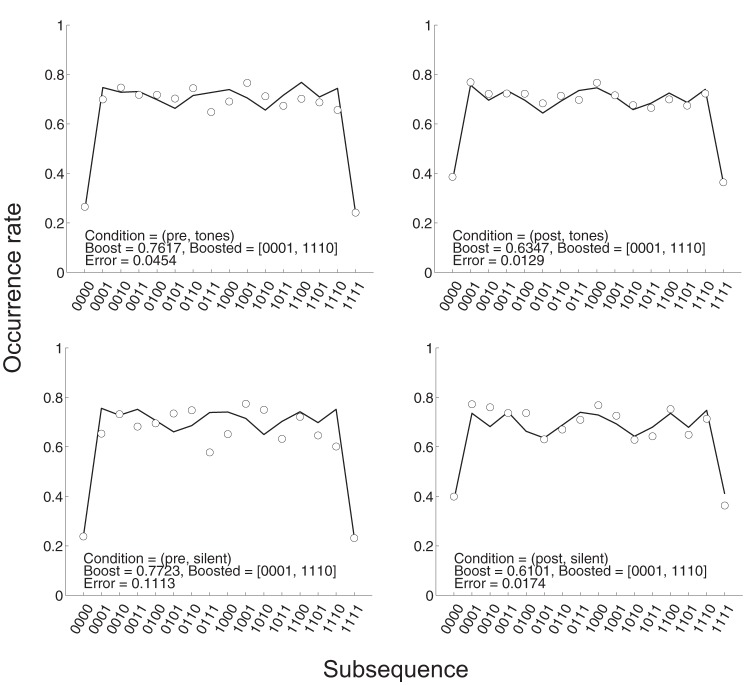
Occurrence rate data (circles) and fits (lines) based on boosting 0001 and 1110 across the four experimental conditions.

**Figure 12 fig12:**
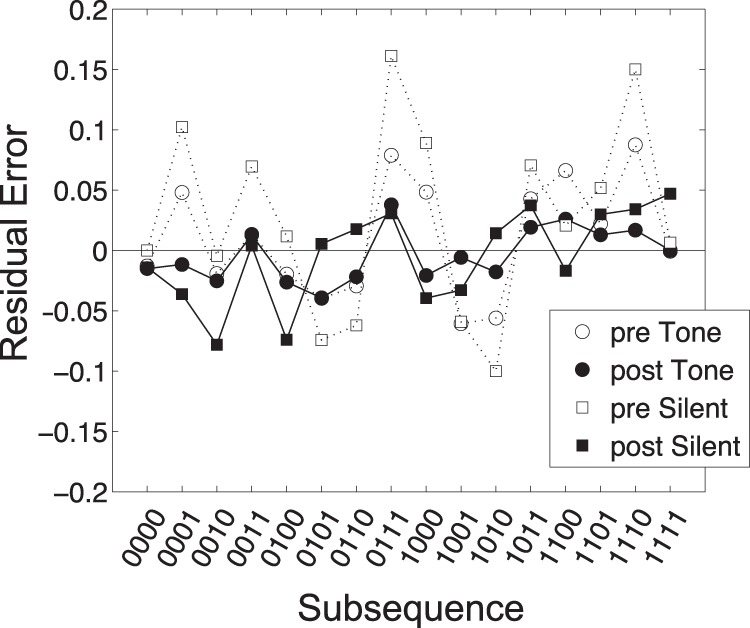
Residual errors from fits shown in Figure 11 when boosting AAAB (0001 and 1110) subsequences. Note that errors are markedly reduced in the postexperience conditions.

The resultant fits are illustrated in Figure 11 and the associated residual errors across generated subsequences are illustrated in Figure 12. Note first that the fits are generally quite good but appear considerably better, with smaller residuals, in the postexposure conditions, suggesting that some learning has taken place. Furthermore, note that the fitted value of β is higher in the both pre-exposure generation conditions (β = 0.76, with tones; β = 0.77, without tones) than the postexposure generation conditions (β = 0.63, with tones; β = 0.61, without tones). This result suggests that postexposure participant generated sequences are closer to what would be expected from a genuinely random source.[Fig-anchor fig11][Fig-anchor fig12]

In Tables 1 and 2 we summarize the results of fitting a range of other models with one free parameter but in which we boosted one of the other seven different pairs of complementary subsequences. Note that (e.g.) boosting the pair 0001 and 1110 (β > 0.5) is equivalent to de-boosting (β < 0.5) the pair (0000 and 1111) so the values of β for these cases sum to 1 (see Table 1) and the residual errors are very similar (see Table 2).[Table-anchor tbl1][Table-anchor tbl2]

Note in Table 2 that the best fits (lowest average residual error) to the human data are obtained by boosting 0001 and 1000 (although boosting 1001 and 0110, which both break a run of length 3 is almost as good). Consequently we propose that, consistent with the key characteristic of the Hahn and Warren account raised above, the best fits to human data are obtained when runs are treated differently from other subsequences.

## General Discussion

### Summary

The purpose of the present study was to investigate the theoretical account of randomness perception put forward by Hahn and Warren (2009). In particular we wanted to go beyond the standard account that presents a picture of randomness perception as highly biased because the frequencies of human-generated subsequences depart from those expected from a truly random process (Figure 1A). While we do not deny that human behavior does not correspond perfectly with the sequences generated by a genuinely random source we suggest that the extent of this departure depends to a large part on the metrics chosen to compare behavior. We present a set of alternative analyses of our data across two experiments and for which human performance is remarkably similar to that of a random process. Furthermore we suggest that the metrics which arise provide a more appropriate means for comparison in taking into account the nature of human experience. We go on to develop a simple model with one free parameter, which implements key characteristics of the Hahn and Warren (2009) account and that generates sequences that match the properties of human generated sequences.

### Mimicry or Genuine Sensitivity to More General Properties of Experienced Random Sequences?

One potential reply to this study might be that it probes (and evaluates) “mimicry”, rather than people’s conception of randomness (on the contrast between conceptions and perceptions of randomness see also, Zhao, Hahn, & Osherson, 2014). Here, it is worth bearing in mind that the majority of studies on random sequence generation have instructed participants to “imagine an unbiased coin” and “generate sequences like it” or “representative” of it (e.g., Kareev, 1992; Nickerson & Butler, 2009; see also Bar-Hillel & Wagenaar, 1991 for an overview). There is good reason for this in that research on intuitive statistics, to which randomness research has always belonged (see, e.g., Tversky & Kahneman, 1974), is not concerned with people’s metalevel explications of statistical concepts (that would amount, in effect, to probing their mathematical knowledge), but rather with intuitive statistical notions implicit in behavior. In the case of randomness, such an intuitive understanding must necessarily derive from experience, and it is the point of recent theoretical accounts such as that of Hahn and Warren (2009) and the empirical work described here to make clear just how much observed behavior may actually resemble people’s experience. Nevertheless, our study does intentionally depart from other sequence generation studies in the past by providing participants with experience of a model random process.

However, a simple analysis (see Figure 10), shows that the participant-generated sequences obtained in Experiment 2 were considerably less well correlated with the specific observed sequence than generic sequences generated by a truly random process. This result suggests that any experiential learning that did take place was unlikely to be simple mimicry. Furthermore, in Experiment 2 we probed participant behavior both before and after exposure to experience so we could assess the extent to which perception was affected. Indeed there was a clear effect of seeing output from a “machine generating a random sequence” that was viewed passively without any need to engage with the sequence (e.g., through outcome prediction as in Edwards, 1961): After exposure, participant-generated sequences were significantly closer to those generated by the random source. Taken together these results suggest that although recent experience does play a role in shaping current perception of randomness, as reflected in a generation task, these effects are not based on the ability to both acquire and reflect faithfully the distributional characteristics of the specific sample sequence seen in the lab. Instead we suggest that even from the relatively short, passive exposure, participants were genuinely sensitive to more general properties of random sequences that were then reflected in their outputs. Based on this result we suggest both that experience of randomness is key to subsequent perception, and that, consequently, the much-bemoaned “biases” in randomness perception and generation are ultimately transient phenomena.

### Metrics to Assess Bias in Randomness Perception

A key result of this article is that the correspondence between human and unbiased theoretical data depends on the statistics used to parameterize performance (and this holds regardless of whether the human data has substantially been altered by the experiment itself). We have presented several analyses that emphasize the similarities. Moreover, these analyses are appropriate in that they reflect the manner in which we are likely to experience random events because of the constraints imposed on human cognition—that is, as a sliding window moving one outcome at a time through a longer but finite sequence of unfolding events. The results presented confirm the argument made in Hahn and Warren (2010) that the mean (expected value) is not an appropriate statistic to characterize the distribution of subsequences generated by either a human or unbiased process under a sliding window analysis. The level of skew in the data is high and it is precisely for such distributions that the median and/or mode are preferable. As noted in Hahn and Warren (2010), it would seem problematic to conclude that average income was $100,000 per month in a population where most made $1,000 and very few made $1,000,000. By the same logic, based on the distributions presented in Figure 2, it is not sensible to suggest that one would expect to see (on average) about one instance of HHHH in 20 coin flips. In contrast the median (Figures 4 and 8) and or/mode (Figures 3 and 9) statistics are more meaningful, and, based on these statistics humans look rather well matched to the genuinely unbiased process.

### Cognitive Constraints

The fact that human and unbiased sequence generation processes share common features for Analysis 2 (at a range of plausible window lengths; see supplementary materials) suggests that it is possible that on average our participants were behaving similarly to the process described in Hahn and Warren (2009) with sliding window length around 4. In practice, individuals are likely to have different and possibly nonstationary sliding window lengths. If enough data is generated, it may be possible to establish a link between individual sequence statistics and a proxy measure of window length such as digit-span or STM capacity. An investigation of this possibility will form the basis of future work.

### A Generative Model of Human Random Sequence Generation

In Section 4 we presented a very simple generative model of how humans might produce random sequences. Nonetheless, this model provides a good description of observed human generation data (see Figure 10) and this is particularly the case for data generated postexposure to the genuinely random source (see Figure 10). Better fits to the data could, of course, be obtained by boosting multiple subsequences or boosting subsequences at multiple lengths. We have chosen not to do this, in part because it would be difficult to choose between such models without extensive data. In addition, the fact that a model that departs rather subtly from a genuinely random generation process captures human behavior so well emphasizes the extent to which characterizing human performance as flawed is potentially unjustified. This is especially true given the way in which the model departs from an unbiased process (i.e., by boosting runs) actually reflects a genuine statistical feature of such sequences under a compelling model of how humans might actually experience an unfolding sequence of random events.

### Generation Tasks Versus Other Randomness Perception Tasks

In the beginning of the article we noted that other tasks (i.e., not involving sequence generation) have been used previously to investigate randomness perception. In first instance, then, our findings are limited to the context of sequence generation. However, sequence generation has been by far the most common task used in this literature and there is evidence that performance in another commonly used task (random sequence judgment; e.g., see Falk & Konold, 1997) is compatible with that in generation tasks (e.g., see Farmer, Warren, & Hahn, 2017). Also, other tasks such as the ingenious (although more indirect) memory-based studies used by Olivola and Oppenheimer (2008) are arguably reliant on the fact that biases in perception have been observed previously in more direct tasks such as sequence generation and judgment. Consequently, the limitation to sequence generation is arguably less restrictive than it might first seem. More important, however, the specific task used is secondary to the major thrust of this article, which is aimed at the question of suitable *metrics* for assessing these bias phenomena in the first place, an issue that is orthogonal to that of the method used to observe such effects.

### So, Is Randomness Perception Biased?

No evidence we present in the present manuscript can argue against the clear departures of human behavior from that which might be expected from an idealized information processing system. Under that definition, then, it is clearly the case that human randomness perception is biased. It is also the case (as noted above) that such departures can have important implications (e.g., see Toneatto et al., 1997). However, our contention, both in Hahn and Warren (2009) and the present study, is that this bias is a natural consequence of the cognitive constraints identified and actually reflects an entirely appropriate tuning for the statistics of the environment as experienced *under those constraints*. In that sense, then, it seems problematic to characterize this behavior merely as a failing.

This point seems all the more important because given enough resolution, deviations between actual human and idealized, optimal performance seem inevitable (e.g., see Jarvstad, Hahn, Rushton, & Warren, 2013). This makes it more fruitful to investigate why specific deviations are observed. It is worth noting here a distinction between the Judgment and Decision Making (JDM) and Vision Science literatures. Visual illusions are not generally referred to as perceptual biases. Papers published in that literature do not generally start out with an emphasis on, and description of, how biased the system is. Rather, illusions are more likely to be discussed as unavoidable side effects of the constraints operating on the system and treated as an opportunity to identify those constraints to explain the behavior. This was once a widely held view in the cognitive literature also; indeed, much of Tversky and Kahneman’s original work on “heuristics and biases” explicitly drew out the methodological parallel to the study of perceptual illusions (Tversky & Kahneman, 1974). However, subsequent decades have arguably witnessed more negative framing of such deviations, and an increased emphasis on bias as an indicator of human cognitive frailty (for a historical overview of bias and its role in psychological research see Hahn & Harris, 2014).

We think the present results illustrate why a return to the perspective of Vision Science would be fruitful when it comes to considering randomness perception. Indeed recent results indicate the importance of not overemphasizing cognitive bias in the JDM literature more generally. A number of recent studies have suggested that when appropriate cognitive constraints are taken into account, and participants engage in well-defined tasks, their behavior is close to optimal (Howes, Warren, Farmer, El-Deredy, & Lewis, 2016; Jarvstad et al., 2013; Jarvstad, Rushton, Warren, & Hahn, 2012; Maloney, Trommershauser, & Landy, 2007; Warren, Graf, Champion, & Maloney, 2012). Furthermore, recent reappraisals of what, on first inspection, appears as irrefutable evidence of cognitive bias in JDM have shown that such behavior might actually be rational when information processing is corrupted by noise (Costello & Watts, 2014; Howes et al., 2016).

## Conclusion

We provide experimental data that is consistent with the account put forward by Hahn and Warren (2009, 2010). Based on the experimental and theoretical work presented here, together with recent related work testing predictions of the Hahn and Warren (2009) account for both random sequence generation and judgment (Farmer et al., 2017), we suggest that apparent biases in human randomness perception should be reevaluated. In particular we suggest that it is problematic to suggest human behavior is flawed simply because it departs from that of an unbiased process on metrics that may not reflect cognitive and task constraints.

## Supplementary Material

10.1037/xhp0000462.supp

## Figures and Tables

**Table 1 tbl1:** Boost Parameters Obtained by Fitting Procedure

Condition	0000 1111	0001 1110	0010 1101	0011 1100	0100 1011	0101 1010	0110 1001	0111 1000
(pre, silent)	.2277	.7723	.5833	.4167	.3529	.6475	.6632	.3368
(post, silent)	.3893	.6101	.5038	.4957	.5000	.4984	.5287	.4706
(pre, tone)	.2383	.7617	.5663	.4339	.3958	.6033	.6170	.3830
(post, tone)	.3652	.6347	.5267	.4714	.4577	.5459	.5362	.4622

**Table 2 tbl2:** Residual Errors Obtained by Fitting Procedure

Condition	0000 1111	0001 1110	0010 1101	0011 1100	0100 1011	0101 1010	0110 1001	0111 1000
(pre, silent)	.1113	.1113	.1748	.1749	.1405	.1405	.0760	.0760
(post, silent)	.0174	.0174	.0356	.0347	.0362	.0364	.0318	.0318
(pre, tone)	.0454	.0454	.1191	.1190	.1120	.1120	.0637	.0638
(post, tone)	.0130	.0130	.0318	.0321	.0322	.0323	.0289	.0290
Average	.0468	.0468	.0903	.0901	.0803	.0803	.0501	.0501

## Footnotes

1 Human participants are prone to overalternation (assumed to be indicative of the gambler’s fallacy) in both the perception and generation of sequences that involve mechanical random devices, such as coins or roulette wheels. They also seem to attribute less alternation than may be empirically justified in the context of fluctuating human performance, displaying the so-called ‘hot hand fallacy’ (see e.g., Ayton & Fischer, 2005).

2 In Experiment 2 we investigate the impact of the auditory stimulus over and above the visual stimulus.

3 Placing emphasis on data beyond *k* = 5 is problematic for other reasons. Note that the number of subsequences to consider is doubled for each increment in *k*. As a consequence the amount of data we have available for each subsequence is reduced. Perhaps more importantly, the amount of data that would be required for a human to reliably discriminate and/or obtain useful summary statistics for *k* > 5 subsequences would be huge (see Hahn & Warren, 2009).
