# Supplementary material for: A Re-Examination of “Bias” in Human Randomness Perception
Source: J Exp Psychol Hum Percept Perform. 2017 Oct 23;44(5):663–80. doi: 10.1037/xhp0000462 (PMC5933241; doi:10.1037/xhp0000462)
Supplement: Supplementary file 1 [file Supplementary.pdf]

Supplementary materials for

Assessing the "bias" in human randomness perception

Paul A. Warren[†*], Umberto Gostoli[†], George D. Farmer[†], Wael El-Deredy[†],

Ulrike Hahn[‡]

† School of Psychological Sciences, University of Manchester, Manchester, M13 9PL, UK

Email: paul.warren@manchester.ac.uk. Tel: +44 (0) 161 2757699

‡ Department of Psychological Sciences Birkbeck University of London London, WC1E 7HX, UK

* To whom correspondence should be addressed (paul.warren@manchester.ac.uk)

S1: Expected frequencies for window lengths k = 3, 5, 6

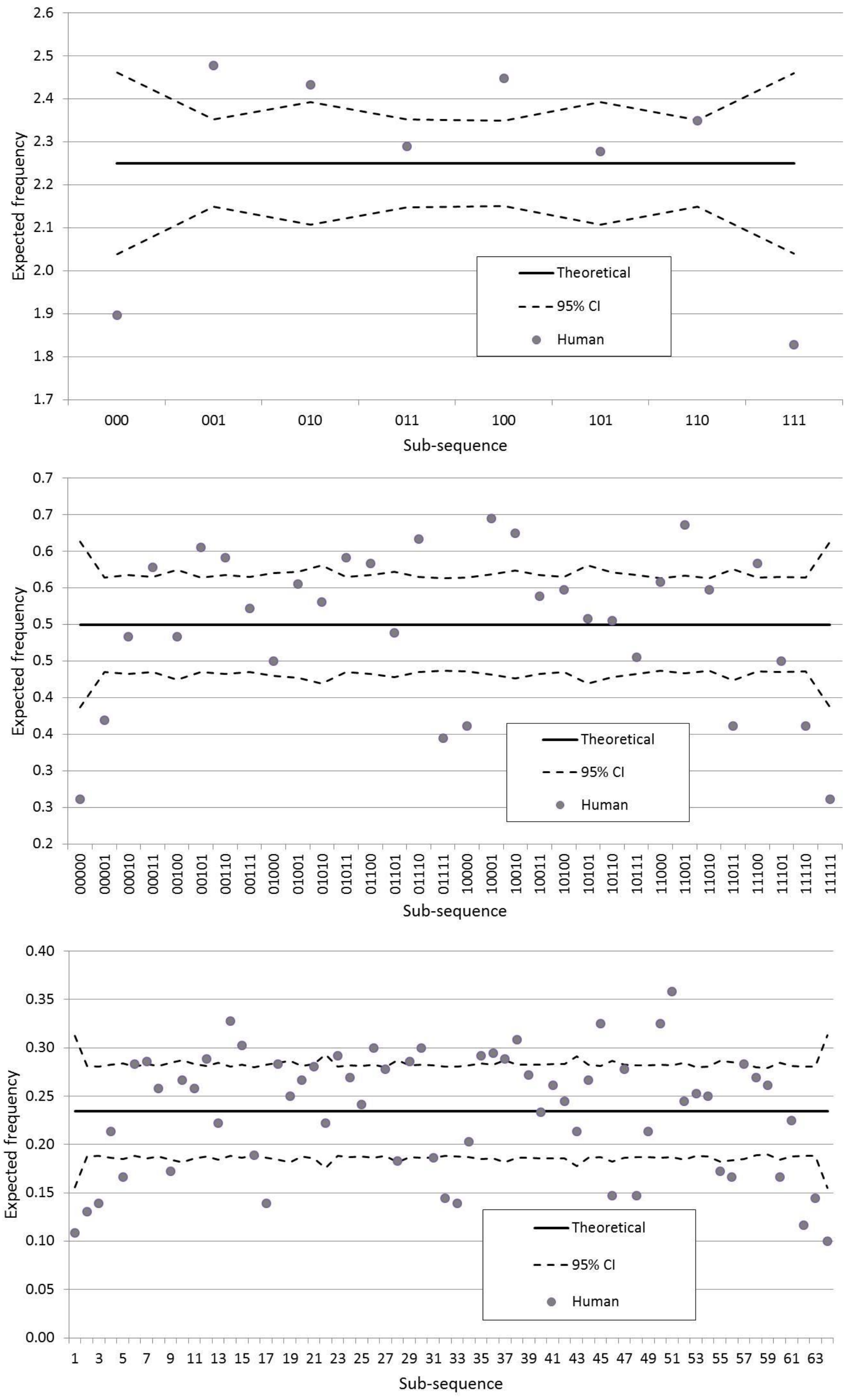

Figure S1: Expected frequencies for varying window length; k = 3 (top panel), k = 5, middle panel, k = 6 (bottom panel). These figures are analogous to those seen in figure1A of the main text.

S2: Occurrence rates for window lengths k = 3, 5, 6

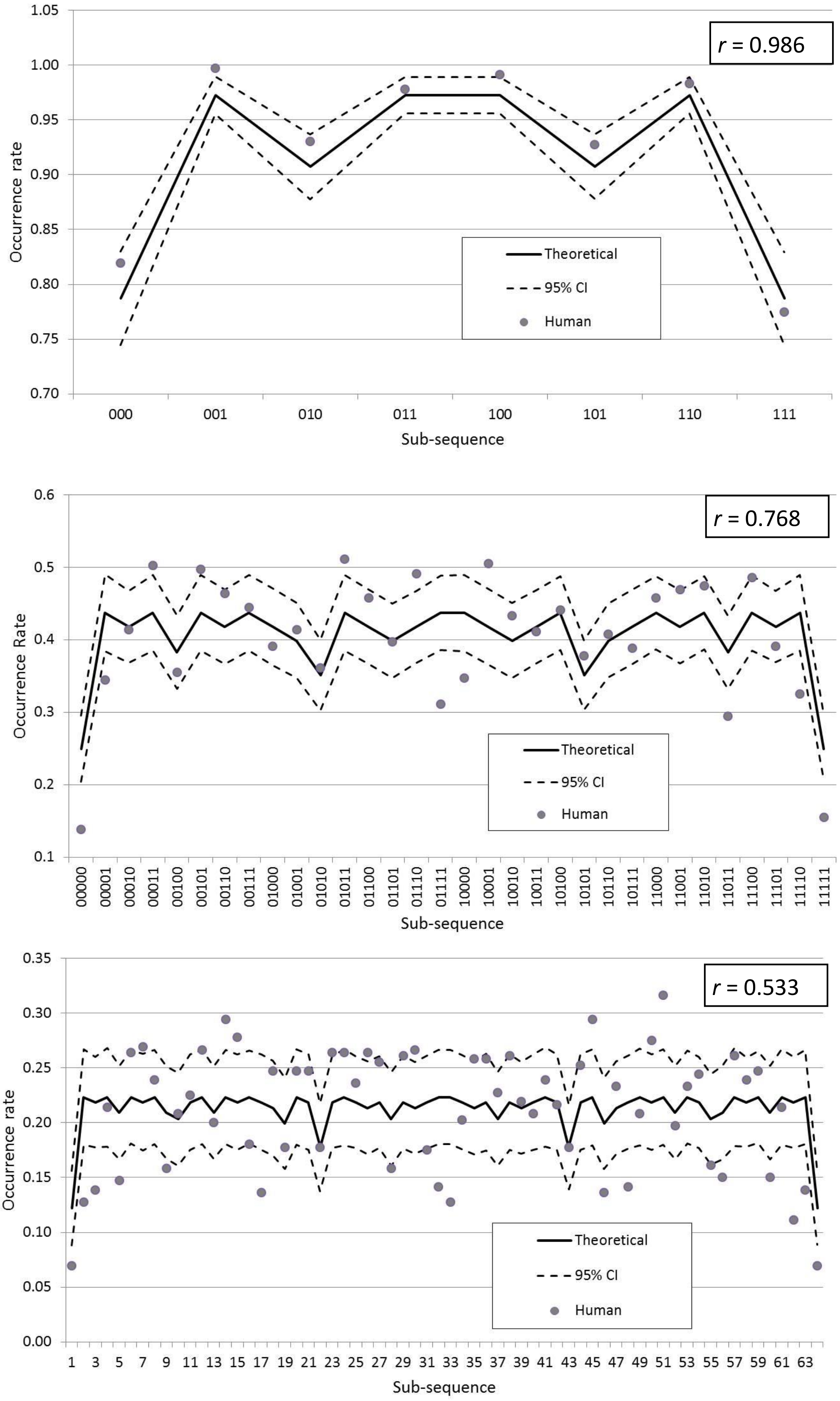

Figure S2: Occurrence rates for varying window length; k = 3 (top panel), k = 5, middle panel, k = 6 (bottom panel). These figures are analogous to those seen in figure1B of the main text.

S3: Correlation between *k* = 4 subsequences in generated and observed sequence

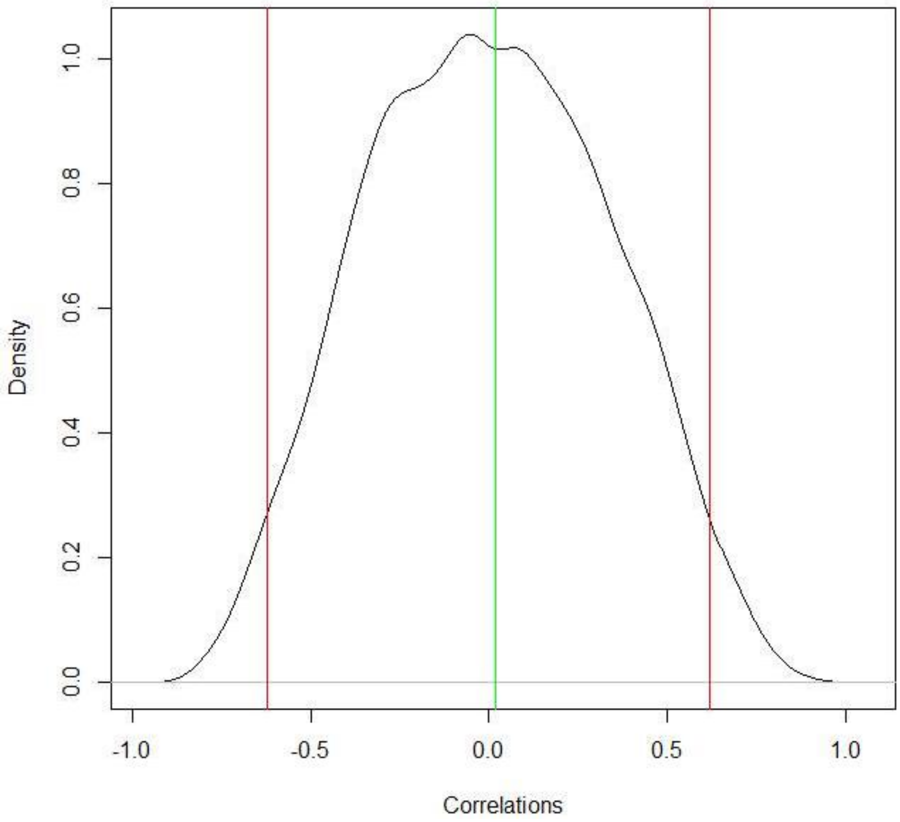

Figure S3: Subsequence frequencies (*k* = 4) *were generated for the 30 x 20 bit sequences observed in the presentation phase. These frequencies were correlated against equivalent frequencies obtained from 1000 more 30 x 20 bit sequences generated by an unbiased random process. The distribution of correlation coefficients is shown in black together with the 95% confidence intervals for the distribution indicated by red vertical lines. For comparison, the green vertical line shows the correlation between the equivalent frequencies for the aggregate participant-generated sequences and those observed in the presentation phase. This analysis suggests that the generated sequences did not have an atypically high correlation with those from the sequence observed in the presentation phase.*
